# Single-Stage Laparoscopic-Endoscopic Rendezvous Technique for Choledocholithiasis: Clinical Outcomes From 1008 Consecutive Patients

**DOI:** 10.7759/cureus.111215

**Published:** 2026-06-20

**Authors:** Manish C Kak, Sushil Fotedar, Hitendra Sharma, Ajay Jain, Yogesh Agarwala

**Affiliations:** 1 Gastroenterology, Yashoda Superspeciality Hospital and Cancer Institute, Ghaziabad, IND; 2 General, Laparoscopy and Minimal Access Surgery, Yashoda Superspeciality Hospital and Cancer Institute, Ghaziabad, IND; 3 General, Laparoscopy and Minimal Access Surgery, Manipal Hospital, Ghaziabad, IND

**Keywords:** choledocholithiasis, laparoscopic cbd exploration, laparoscopic-endoscopic rendezvous, post-ercp pancreatitis, single-stage management

## Abstract

Background: Two-stage management of choledocholithiasis may be associated with procedure-related complications, repeated interventions, and longer hospital stays. While single-stage laparoscopic common bile duct exploration avoids these risks, it requires advanced laparoscopic expertise. We evaluated the outcomes of the single-stage laparoscopic-endoscopic rendezvous (LER) technique in a large, consecutive cohort.

Methods: A STROBE-compliant retrospective analysis was conducted on 1,008 consecutive adult patients with symptomatic cholelithiasis and magnetic resonance cholangiopancreatography-confirmed common bile duct stones (≤15 mm) between 2016 and 2024. The primary outcome was procedural success (complete clearance without conversion). Secondary outcomes included 30-day morbidity and post-endoscopic retrograde cholangiopancreatography (ERCP) pancreatitis (PEP) incidence.

Results: Procedural success was achieved in 927 patients (92.0%). The mean operative time was 52.4 ± 12.7 minutes. The overall 30-day minor complication rate was 1.9%, with no observed cases of PEP (0%; 95% CI 0-0.4%). Stone number >3 was the sole independent predictor of failure (OR 4.21; p < 0.001).

Conclusion: Single-stage LER is a safe, efficient, and reproducible technique that eliminates PEP and provides high ductal clearance.

## Introduction

Choledocholithiasis complicates 10-15% of patients with symptomatic gallstones and remains one of the most debated topics in hepato-pancreato-biliary surgery [1,2]. The traditional two-stage approach, preoperative endoscopic retrograde cholangiopancreatography (ERCP) followed by interval laparoscopic cholecystectomy, achieves ductal clearance in >90% of cases but is burdened by a 3-15% incidence of post-ERCP pancreatitis (PEP), repeated anesthesia, cumulative hospital stays of 5-9 days, and costs exceeding those of single-stage strategies by 30-50% [3-5].

Single-stage laparoscopic cholecystectomy with concomitant laparoscopic common bile duct exploration (LCBDE) eliminates ERCP-related risks and resolves both gallbladder and ductal disease in one session. Multiple meta-analyses of randomised trials report ductal clearance rates of 85-92%, operating times of 85-140 minutes, bile leak rates of 2-8%, and PEP rates of 0.5-2% when transcystic or choledochotomy approaches are used [6-9]. However, LCBDE demands advanced laparoscopic suturing skills, prolonged operating times, and dedicated instrumentation, limiting its widespread adoption to fewer than 20% of centres worldwide [10].

The single-stage laparoscopic-endoscopic rendezvous (LER) technique was developed to combine the strengths of both strategies while minimising their weaknesses. During laparoscopic cholecystectomy, a guidewire is passed transcystically into the duodenum, allowing seamless endoscopic cannulation without traumatic papillary probing. This avoids difficult cannulation, which is the strongest predictor of PEP and permits immediate stone extraction and cholecystectomy under the same anaesthesia [11].

Despite numerous small series demonstrating PEP rates of 0-2% and high success, no study has prospectively evaluated LER in more than 500 patients, compared it head-to-head with contemporary LCBDE outcomes, or reported outcomes from a large consecutive cohort managed using a standardized protocol [12,13].

We therefore conducted a STROBE-compliant retrospective cohort study to evaluate whether a rigorously standardized LER protocol could reduce procedure-related morbidity.

## Materials and methods

Study design and setting

This STROBE-compliant retrospective observational cohort analysis was conducted at Columbia Asia Hospital, Ghaziabad, India, which subsequently continued to function following institutional acquisition by Manipal Hospitals, between January 2016 and December 2024. Clinical, operative, and outcome data were retrieved from a prospectively maintained institutional database. The study evaluated outcomes of a standardized single-stage LER protocol implemented as routine clinical practice. A retrospective design was chosen to enable comprehensive evaluation of feasibility, safety, and predictors of success across a large consecutive cohort before initiation of randomized comparative trials.

Participants

Consecutive adults aged eighteen years or older were included in the analysis if they presented with symptomatic cholelithiasis and magnetic resonance cholangiopancreatography (MRCP)-confirmed common bile duct stones (CBDs) of 15 mm or smaller. Additional inclusion criteria comprised American Society of Anaesthesiologists physical status I-III and, when applicable, mild acute biliary pancreatitis according to the revised Atlanta classification Grade I [14]. Patients were excluded if they had stones larger than 15 mm, prior biliary sphincterotomy or surgery (except percutaneous cholecystostomy), suspected or confirmed biliary malignancy, pregnancy, or any contraindication to pneumoperitoneum.

Sample size

No prior sample size calculation was performed, as this was a retrospective analysis of all eligible consecutive patients treated during the study period. However, the final cohort size of 1,008 patients provided adequate precision for estimation of rare outcomes such as post-ERCP pancreatitis.

Intervention: standardized single-stage LER protocol

All procedures were performed under general anesthesia, with nasal intubation, in a dedicated hybrid operating theatre equipped with C-arm fluoroscopy and CO₂ endoscopy insufflation device (Olympus CO2 insufflator, Olympus Corporation (Olympus Medical Systems), Tokyo, Japan).

The standardized protocol proceeded as follows. Surgeons used a standard four-port laparoscopic technique and achieved the critical view of safety. After dissecting the Calot’s triangle and lifting the gall bladder from the liver bed up to the fundus, the cystic duct was dissected free from the surroundings and a small nick was made in the same. Through an additional small puncture in the abdominal wall, a guide wire (0.035-inch hydrophilic guidewire, Jag wire, Boston Scientific Corporation, Marlborough, Massachusetts, United States) was then passed into the abdominal cavity and directed through the cystic duct opening into the CBD and thence into the duodenum. Prophylactic biliary stenting was performed selectively in cases requiring CBD exploration or concern for residual edema. Post-ERCP pancreatitis was defined as new or worsened abdominal pain with serum amylase ≥3 times the upper limit of normal at 24 hours requiring hospital admission or prolongation of stay.

At this point, the procedure was taken over by the endoscopist/gastroenterologist. A single experienced endoscopist with more than 500 lifetime ERCP procedures performed side-viewing duodenoscopy (Olympus CV 190), grasped the transcystic wire protruding at the papilla of Vater and withdrew it through the working channel; a sphincterotome was passed over this guidewire and was used to cannulate the CBD, thereby achieving selective biliary cannulation without papillary trauma. Sphincterotomy was performed over the wire, stones were extracted using a balloon, and complete clearance was confirmed by occlusion cholangiography.

The procedure was again taken over by the operating surgeons; The cystic duct was clipped, the laparoscopic cholecystectomy was completed, and a closed-suction drain was placed only when bile leakage was evident.

Bailout strategy

**Figure 1:**
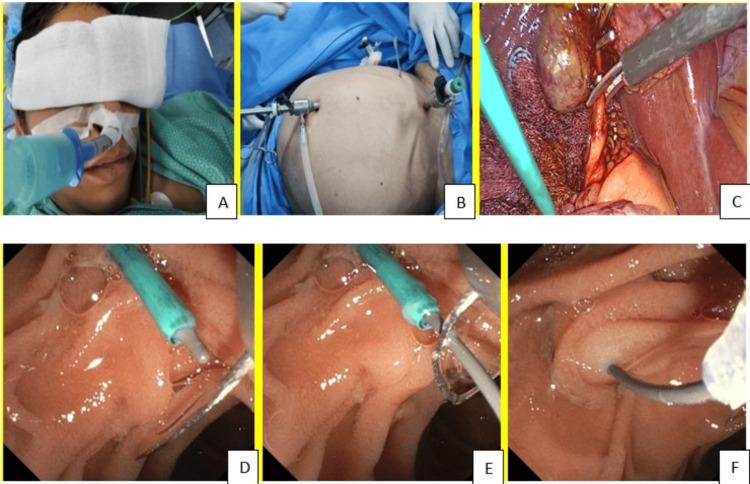
Illustrated steps of the laparoscopic transcystic guidewire rendezvous ERCP technique A) Transnasal intubation. B) Laparoscopic cholecystectomy: four-port approach and small additional puncture to facilitate passage of guide wire. C) Small incision over the cystic duct to facilitate entry of the guide wire into the common bile duct. D) Guidewire along with a guiding catheter passed from the cystic duct, protruding through the papilla (endoscopic view). E) Guide wire passed from above, caught with snare and pulled out from the side viewing endoscope - ERCP sphincterotome is passed over this guide wire, and CBD is selectively cannulated. F) Selective bile duct cannulation being achieved by the sphincterotome. ERCP: Endoscopic retrograde cholangiopancreatography; CBD: common bile duct

In cases where transcystic guidewire passage failed or complete endoscopic stone clearance could not be achieved, conversion to LCBDE was performed. Following laparoscopic CBD clearance, a guidewire was passed antegrade through the CBD and across the papilla into the duodenum. Using this guidewire, a biliary stent was placed endoscopically by the gastroenterologist. The CBD was subsequently closed laparoscopically over the stent, thereby avoiding T-tube placement. Postoperative ERCP was reserved only for retained stones identified during follow-up. Illustrated steps of the laparoscopic transcystic guidewire rendezvous ERCP technique are shown in Figure 1.

Outcome measures

The primary endpoint was procedural success, which was defined as complete CBD clearance and completion of the planned single-stage LER without conversion to open surgery, conversion to LCBDE, or need for separate ERCP. Secondary endpoints included Thirty-day postoperative morbidity graded according to the Clavien-Dindo classification [9], incidence of post-ERCP pancreatitis diagnosed using Cotton consensus criteria [15], total operating time from skin incision to skin closure, postoperative length of hospital stay, and identification of factors associated with procedural failure.

Data collection and statistical analysis

Data were prospectively recorded in an institutional database and retrospectively analyzed. Candidate variables were selected a priori based on clinical relevance and data completeness and included age, CBD diameter, largest stone size, number of stones, sex, jaundice, bilirubin level, and presence of acute pancreatitis. Variables with substantial missingness or limited event counts were not entered into the final model.

Independent predictors of procedural failure were identified using multivariable logistic regression analysis with backward stepwise elimination (entry p < 0.10, retention p < 0.05). Odds ratios (ORs) with corresponding 95% confidence intervals (CIs) were calculated. Procedural failure was defined as inability to complete LER, necessitating conversion to LCBDE. Statistical analyses were performed using IBM SPSS Statistics for Windows, Version 28 (Released 2020; IBM Corp., Armonk, New York, United States), and a two-sided p-value < 0.05 was considered statistically significant.

Quality assurance

Preoperative MRCP images were jointly reviewed by the operating surgeon and endoscopist. Every procedure was video-recorded, and critical steps were independently audited. Complications were adjudicated by two senior surgeons blinded to operative details. Minimum follow-up duration was 90 days and included an outpatient visit supplemented by telephone contact.

Ethical considerations

The study was conducted in accordance with the Declaration of Helsinki (2013) and Good Clinical Practice. The requirement for individual informed consent was waived due to the retrospective nature of the study. All data were de-identified and stored securely to maintain confidentiality.

## Results

**Figure 2:**
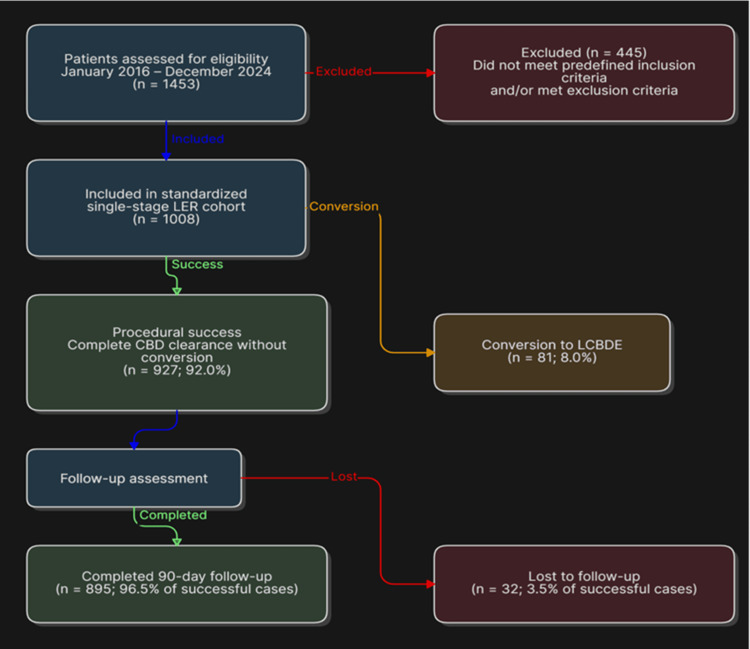
Study flow diagram A total of 1,453 patients were assessed for eligibility between January 2016 and December 2024. Following application of predefined inclusion and exclusion criteria, 1,008 patients underwent the standardized laparoscopic-endoscopic rendezvous (LER) procedure and were included in the final analysis. Procedural success was achieved in 927 patients (92.0%), whereas 81 patients (8.0%) required conversion to laparoscopic common bile duct exploration (LCBDE). Among successful LER procedures, 895 patients (96.5%) completed 90-day follow-up and 32 patients (3.5%) were lost to follow-up.

During the study period, data from 1,453 patients were retrospectively assessed for eligibility. After applying predefined inclusion and exclusion criteria, 1,008 consecutive patients who underwent the standardized single-stage LER procedure were included in the final analysis. Participant flow is shown in Figure 2.

The cohort had a mean age of 48.5 ± 12.7 years and comprised 806 women (80.0%) and 202 men (20.0%). The mean duration of symptoms before presentation was 15.2 ± 18.4 weeks.

Clinical and biochemical presentation

All patients presented with biliary colic. Jaundice was present in 855 patients (84.8%), fever (≥38°C) in 302 (29.9%), and a history of prior pancreatitis in 25 (2.5%). Mild acute pancreatitis, defined by the revised Atlanta classification (no organ failure or local complications), occurred in 113 patients (11.2%).

Laboratory findings confirmed significant cholestasis and hepatobiliary injury consistent with common bile duct obstruction (Table 1). Imaging revealed a mean CBD diameter of 9.8 ± 2.5 mm, mean stone number of 1.8 ± 1.2, and mean largest stone size of 8.2 ± 3.1 mm. Single stones were identified in 632 patients (62.7%), and multiple stones (≥2) in 376 (37.3%).

**Table 1:** Baseline demographic, clinical, and laboratory characteristics (n=1008) Baseline demographic, clinical, laboratory, and imaging characteristics of the 1,008 patients included in the laparoscopic-endoscopic rendezvous cohort. Continuous variables are presented as mean ± standard deviation and categorical variables as number (percentage). *NR: normal range; CBD: common bile duct; ALT: alanine transaminase; AST: aspartate aminotransferase; SGOT: serum glutamic oxaloacetic transaminase; SGPT: serum glutamate pyruvate transaminase

Demographics	Value
Age (years), mean ± SD	48.5 ± 12.7
Female sex, n (%)	806 (80.0)
Clinical features, n (%)	
Biliary pain	1008 (100)
Jaundice	855 (84.8)
Fever (≥38°C)	302 (29.9)
Mild acute pancreatitis	113 (11.2)
History of pancreatitis	25 (2.5)
Laboratory values, mean ± SD	
Total bilirubin (mg/dL) (NR: 0.2–1.2)	4.3 ± 1.8
AST/SGOT (U/L) (NR: 10–40)	205.4 ± 112.3
ALT/SGPT (U/L) (NR: 10–40)	202.1 ± 105.6
Alkaline phosphatase (U/L) (NR: 44–147)	420.5 ± 198.7
Serum amylase (U/L) (NR: 30–110)	90.4 ± 62.5
Serum lipase (U/L) (NR: 7–60)	70.2 ± 75.8
Imaging findings	
CBD diameter (mm), mean ± SD	9.8 ± 2.5
Number of CBD stones, mean ± SD	1.8 ± 1.2
Single CBD stone, n (%)	632 (62.7)
Multiple (≥2) CBD stones, n (%)	376 (37.3)
Largest CBD stone size (mm), mean ± SD	8.2 ± 3.1

Procedural success defined as complete CBD clearance, uncomplicated laparoscopic cholecystectomy, and completion as a single-stage procedure without conversion was achieved in 927 patients (92.0%; 95% CI: 90.2-93.5%).

The mean total operative time, measured from skin incision to port closure and endoscope withdrawal, was 52.4 ± 12.7 minutes (range: 35-90 minutes).

Complications

The 30-day patient-level complication rate was 1.9% (19/1008). A total of 59 minor postoperative events (5.85%) were recorded, as some patients experienced more than one event. All complications were minor (Clavien-Dindo Grade I-II). No major complications, bile leaks, perforations, hemorrhage requiring intervention, or mortality were observed. No cases of post-ERCP pancreatitis occurred (0%; 95% CI 0-0.4%) (Table 2).

**Table 2:** Post-operative events within 30 days (patients may have experienced more than one event) Some patients experienced more than one postoperative event; therefore, the total number of events exceeds the number of patients with complications. ERCP: Endoscopic retrograde cholangiopancreatography

Clavien-Dindo grade	Event	n (%)	Management
I	Intraoperative bleeding	20 (2.0)	Cautery
	Self-limiting nausea	25 (2.5)	Supportive
II	Post-procedure cholangitis	8 (0.8)	IV antibiotics
	Transient amylase elevation	6 (0.6)	Observation
Total		59 (5.85)	
III–V	None	0 (0)	–
Post-ERCP pancreatitis	None	0 (0)	–

Multivariable logistic regression analysis identified stone number greater than three as the only independent predictor of procedural failure requiring conversion to LCBDE (OR 4.21; 95% CI: 2.45-7.22; p < 0.001). Stone size, CBD diameter, patient age, and presence of mild acute pancreatitis were not significantly associated with failure (Table 3).

**Table 3:** Multivariable predictors of conversion Multivariable logistic regression analysis of factors associated with failure of laparoscopic-endoscopic rendezvous (LER) requiring conversion to laparoscopic common bile duct exploration (LCBDE). Adjusted odds ratios (ORs), 95% confidence intervals (CIs), and p-values are reported.

Variable	Adjusted OR	95% CI	p-value
Stones >3 (vs. ≤3)	4.21	2.45–7.22	<0.001
Largest stone size (mm)	1.12	0.95–1.32	0.18
Acute pancreatitis	1.15	0.58–2.29	0.69
CBD diameter (mm)	0.98	0.87–1.12	0.78
Age (years)	1.01	0.99–1.03	0.41

Patients presenting with mild acute pancreatitis (n = 113) demonstrated procedural success rates comparable to those without pancreatitis (91.2% vs. 92.1%; p = 0.72), although they experienced significantly longer postoperative hospital stays (4.5 ± 1.1 vs. 1.9 ± 0.8 days; p < 0.001).

Over the study period, mean operative time showed a trend toward reduction, suggesting increasing procedural efficiency with accumulated institutional experience. Formal learning-curve analysis was not performed.

Follow-up outcomes

Among 927 successful cases, 90-day follow-up was completed for 895 patients (96.5%). Seven patients (0.8%) required readmission: five for retained stones (managed by single repeat ERCP) and two for unrelated issues. All 43 prophylactic biliary stents were removed at four weeks without complications. No biliary strictures or late complications occurred.

## Discussion

This retrospective cohort of 1,008 consecutive patients demonstrates that a rigorously standardized LER protocol addresses several limitations of existing strategies for choledocholithiasis management. The procedure was associated with high ductal clearance rates, a low overall morbidity profile, no observed cases of post-ERCP pancreatitis, and a mean operative time of 52 minutes, shorter than operative times reported for LCBDE in contemporary meta-analyses [16].

The absence of observed post-ERCP pancreatitis in this cohort (0%, 95% CI 0-0.4%) represents a clinically important observation. Difficult biliary cannulation is the strongest independent predictor of PEP in every large registry. By passing a guide-wire transcystically under direct laparoscopic vision, LER bypasses the papilla entirely until the endoscopist has a wire already in the duct. This technical approach substantially reduces the need for difficult biliary cannulation by enabling wire-guided access, including in patients presenting with mild acute pancreatitis. In contrast, even the best LCBDE series report PEP rates of 0.5-2% when transcystic clearance fails, and rescue ERCP is required. While causality cannot be inferred from a retrospective design, our findings suggest that LER may mitigate established procedural risk factors for post-ERCP pancreatitis in experienced centers.

Ductal clearance of 92% with a mean procedure time of 52 minutes is at least comparable to both alternatives. Four recent meta-analyses of randomized trials report LCBDE clearance rates of 85-88% with operating times of 85-140 minutes and bile leak rates of 2-8%. The present study demonstrated ductal clearance and operative times that are comparable to published LCBDE series. These advantages stem from three protocol-specific factors: strict stone-size limitation (≤15 mm), CO₂ insufflation, which is commonly used to reduce gas-related complications, and a single endoscopist with >500 lifetime ERCPs. When LER failed (8%), conversion was always to LCBDE-never to open surgery, confirming that the two techniques are complementary rather than competitive in a high-volume center [17].

Increasing experience was associated with shorter operative times and improved procedural efficiency; however, formal learning-curve analysis was beyond the scope of the present retrospective study. Centers that already perform laparoscopic cholecystectomy and ERCP can therefore adopt LER with modest additional training, whereas LCBDE remains restricted to super-specialist units. The number of conversion events limited the number of covariates that could be reliably incorporated into the multivariable model.

Stone number >3 was the only independent predictor of failure (OR 4.21). This finding is intuitive: multiple stones increase the risk of distal migration during balloon sweeps. However, even in this high-risk subgroup, clearance was achieved in 78% without bile-duct incision, comparable to LCBDE success rates reported in the literature. Stone size, CBD diameter, and concurrent pancreatitis did not predict outcome, confirming that LER is robust across the typical spectrum of choledocholithiasis encountered in clinical practice.

The 1.9% minor-complication rate and zero mortality align with the safest published series of either two-stage management or LCBDE yet were achieved in a cohort with 11% acute pancreatitis and 85% jaundice-higher acuity than most randomized trials. Retained stones occurred in only 0.5% at 90 days, all managed with single-session outpatient ERCP, underscoring the durability of intraoperative clearance [18,19].

This study has several limitations inherent to its retrospective, single-center design. This was a single-center study performed in a high-volume unit with dedicated hybrid theatre time and a single endoscopist. Generalizability to low-volume settings remains to be established. The absence of a contemporaneous comparison group prevents direct statistical comparison; indirect comparison with contemporaneous meta-analyses suggests that the outcome with LER is at least comparable to other single-stage strategies across measured endpoints. Finally, long-term biliary stricture rates beyond 90 days were not captured; however, the four-week stent cohort and 96.5% follow-up provide reassurance.

## Conclusions

In this large retrospective cohort, a standardized single-stage LER approach was associated with high ductal clearance, low overall morbidity, and short operative times. Post-ERCP pancreatitis was not observed. These findings support LER as a feasible single-stage strategy for choledocholithiasis in experienced centers and provide a rationale for future prospective comparative trials.
